# Association between Pericarotid Fat Density and Positive Remodeling in Patients with Carotid Artery Stenosis

**DOI:** 10.3390/jcm13133892

**Published:** 2024-07-02

**Authors:** Daina Kashiwazaki, Shusuke Yamamoto, Naoki Akioka, Emiko Hori, Kyo Noguchi, Satoshi Kuroda

**Affiliations:** 1 Department of Neurosurgery, Graduate School of Medicine and Pharmaceutical Sciences, University of Toyama, Toyama 930-0194, Japan; shuyama@med.u-toyama.ac.jp (S.Y.); akioka@med.u-toyama.ac.jp (N.A.); emihori@med.u-toyama.ac.jp (E.H.); skuroda@med.u-toyama.ac.jp (S.K.); 2 Department of Radiology, Graduate School of Medicine and Pharmaceutical Sciences, University of Toyama, Toyama 930-0194, Japan; kyo@med.u-toyama.ac.jp

**Keywords:** carotid stenosis, density, inflammation, pericarotid fat, positive remodeling, vulnerable plaque

## Abstract

**Background/Objectives**: The underlying mechanism of the potential involvement of inflammatory crosstalk between pericarotid fat and vascular layers in atherosclerosis pathogenesis is unclear. We investigated the association between pericarotid fat density and positive remodeling and inflammatory markers in carotid stenosis. We hypothesized that pericarotid fat density might serve as a marker of plaque inflammation in a clinical setting. **Methods**: We evaluated the stenosis degree and pericarotid fat density in 258 patients with carotid plaques. Plaque composition was examined, and the correlation between pericarotid fat density and expansive remodeling was investigated. Pearson’s product–moment correlation coefficient was used to examine the relationship between pericarotid fat density and the expansive remodeling ratio. We also evaluated the relationship of pericarotid fat density with plaque composition, degree of stenosis, and macrophage and microvessel counts by. The subgroup analysis compared these factors between symptomatic mild carotid stenosis. **Results**: The pericarotid fat density was −63.0 ± 11.1 HU. The carotid fat densities were −56.8 ± 10.4 HU in symptomatic and −69.2 ± 11.4 HU in asymptomatic lesions. The pericarotid fat density values in intraplaque hemorrhage, lipid-rich necrotic core, and fibrous plaque were −51.6 ± 10.4, −59.4 ± 12.8, and −74.2 ± 8.4 HU, respectively. Therefore, the expansive remodeling ratio was 1.64 ± 0.4. Carotid fat density and expansive remodeling ratio were correlated. Immunohistochemistry showed high macrophage and microvessel levels (143.5 ± 61.3/field and 121.2 ± 27.7/field, respectively). In symptomatic mild carotid stenosis, pericarotid fat density was correlated with other inflammatory factors. The pericarotid fat density and expansive remodeling ratio (2.08 ± 0.21) were high in mild stenosis (−50.1 ± 8.4 HU). **Conclusions**: Pericarotid fat and intraplaque components were well correlated. Carotid fat density may be a marker of plaque inflammation in carotid plaques.

## 1. Introduction

Despite steady advances in medical care, ischemic stroke is the leading cause of mortality and neurological disability worldwide, and atherosclerotic carotid artery disease accounts for 15–20% of strokes [1,2]. Atherosclerotic carotid plaques are major contributors to ischemic stroke. Atherosclerotic plaque rupture can lead to distal embolism, which is caused by thrombus formation and embolization into the distally located intracranial cerebral arteries [3]. An important risk factor for the ischemic event is the presence of vulnerable plaques in the carotid arteries [4,5,6]. Various radiological markers have been described for detecting vulnerable plaques. Intraplaque hemorrhage (IPH), lipid-rich necrotic core (LR/NC), plaque ulceration, and thin or ruptured fibrous cap increase plaque vulnerability to rupture and are related to ischemic events [7,8,9]. Recent studies have suggested that expansive arterial remodeling is also related to plaque vulnerability [10,11]. Increasing evidence suggests that inflammation plays an important role in the development of plaque vulnerability and that vulnerable plaques can activate various immune processes [12,13]. Vascular inflammation drives plaque formation and is a typical characteristic of plaque rupture, which leads to ischemic stroke. In particular, inflammatory crosstalk between pericarotid fat and vascular layers may contribute to the pathogenesis of atherosclerosis [14,15,16]; however, the underlying causal mechanism relating pericarotid fat to the development of vulnerable plaques remains unclear. To further elucidate the mechanism underlying increased pericarotid fat density, we hypothesized that increased pericarotid fat density, which is a marker of vulnerable atherosclerotic plaques, is associated with positive remodeling. Furthermore, pericarotid fat density may be a useful tool for detecting plaque vulnerability. Therefore, the primary purpose of this study was to determine the association between pericarotid fat density and positive remodeling in patients with atherosclerotic carotid artery disease. Second, we investigated the specific characteristics driving the potential associations between carotid fat density and positive remodeling ratio. Finally, we confirmed the relationship between pericarotid fat density and inflammatory markers using carotid endarterectomy (CEA) specimens. In this study, we used markers of macrophage and intraplaque vessels because these are inflammation markers that reflect plaque vulnerability [17,18].

## 2. Materials and Methods

### 2.1. Research Ethics

This cross-sectional study was approved by the Review Board in our institution. We analyzed a prospective database of patients treated by CEA/CAS at our institution. Informed consent was obtained from all patients and guardians by the opt-out method on our homepage. In accordance with the ethical standards of the institutional research committees, this study did not require formal consent. Instead, the outline of the study was available to the public on our homepage, and an option for patients to decline inclusion in the research was provided.

### 2.2. Study Population

This was a retrospective, cross-sectional study. The inclusion criterion was patients with carotid plaque who were treated using carotid endarterectomy (CEA) or carotid artery stenting (CAS) at our institution between January 2015 and December 2022. Our treatment strategy primarily recommends the use of CEA for symptomatic and CAS for asymptomatic lesions. However, a crossover of surgical methods was contemplated for patients at risk of surgical complications associated with procedures.

Antiplatelet therapy and statin were performed for patients who underwent CEA. Dual antiplatelet therapy and statin were performed for patients who underwent CAS. Antiplatelet therapy continued during the perioperative period.

Patients with symptomatic mild (<50%) carotid stenosis underwent CEA with recurrent ischemic events despite prior appropriated medical treatment or who were at high risk of further ischemic events due to vulnerable plaques on MR plaque imaging. Patients with asymptomatic mild carotid stenosis were treated by medical treatment without CEA/CAS. Patients with carotid stenosis were classified as symptomatic or asymptomatic. We considered symptomatic patients with transient ischemic attack (TIA), amaurosis, retinal artery occlusion, or ischemic stroke of the cerebral hemisphere. TIA was defined as a brief episode of neurological dysfunction, such as hemiparesis, dysarthria, or dysphagia, without cerebral infarction detected by diffusion-weighted imaging (DWI) sequences in magnetic resonance imaging (MRI). Patients with neurological symptoms detected on MRI-DWI were classified as having ischemic stroke [19]. Patients without neurological symptoms but with silent new ischemic lesions are defined as asymptomatic. Clinical data on demographics, ischemic event type, past history, medical treatment, and risk factors for stroke were collected.

All patients underwent CTA, MRI (DWI), T2-weighted imaging, and fluid-attenuated inversion recovery, and magnetic resonance (MR) plaque imaging.

**Figure 1:**
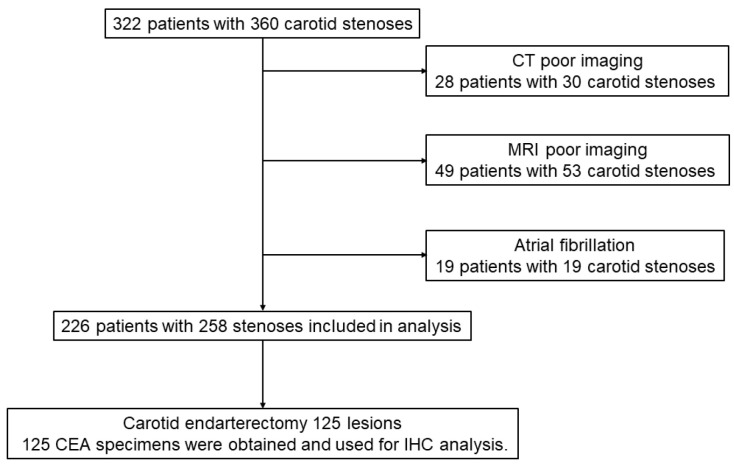
Flow chart of patient selection.

A total of 322 patients with 360 carotid stenoses were treated during the study period; 77 patients with 83 lesions were excluded because of poor radiological imaging, including MR plaque imaging or CTA. Further, 19 patients with 19 carotid stenosis were excluded because of atrial fibrillation. Therefore, 226 patients with 258 carotid plaques were included in this study. Of the 258 lesions, 125 were treated with CEA, while 133 lesions were treated with CAS. Figure 1 shows a flow chart of the patient selection process used in this study.

We measured stenosis degree, pericarotid fat density, plaque composition, and expansive remodeling ratio. The detailed methods for each radiological parameter are described in the following sections.

Patients were excluded if they had carotid stenosis due to radiation, dissection, or restenosis after CEA, CAS, or poor radiological imaging. Further, patients with atrial fibrillation were excluded because of common cause of acute ischemic events due to embolization.

We examined the correlation between pericarotid fat density and the expansive remodeling ratio. We also investigated the relationship between the pericarotid fat density and each plaque composition or stenosis degree. Finally, we evaluated the correlation between pericarotid fat density, CD68-positive macrophage, and CD31-positive microvessel counts from CEA specimens. The subgroup analysis considered these test results according to symptomatic mild carotid stenosis status.

### 2.3. CTA and Pericarotid Fat Density Measurement

All patients underwent dual-energy CT with iodine contrast medium using a third-generation 192-section dual-source computed tomography SOMATOM Force system (Siemens Healthcare GmbH, Forchheim, Germany). To calculate the correct scan timing, a bolus tracking technique was used. The examinations were performed after administering the contrast material. The examination phase was obtained by administering 60–80 mL of prewarmed contrast medium. The coverage extended from the aortic arch to the carotid siphon. The computed tomography technical parameters were as follows: slice thickness, 0.6 mm; matrix size, 512 × 512; and field of view (FOV), 20 cm. The examinations were performed by a physician in the Department of Radiology. The stenosis degree was determined based on these data, following the criteria of the North American Symptomatic Carotid Endarterectomy Trial (NASCET) [20]. Carotid stenosis was classified, according to the stenosis degree, as mild (<50%), moderate (≥50% and <70%), or severe (≥70%).

**Figure 2:**
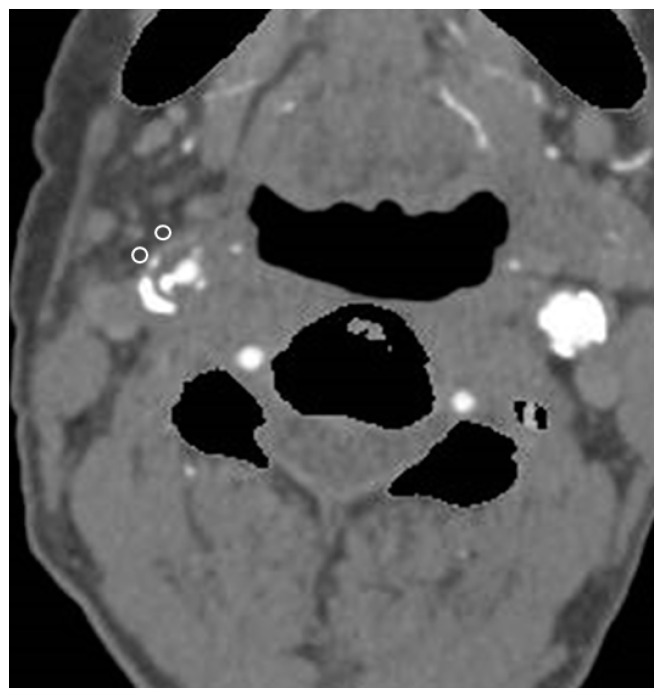
Computed tomography angiography of a 76-year-old man showing right carotid stenosis. Two regions of interest ROIs (white, round) were placed in the pericarotid fat around the carotid plaque. In this case, the 2 ROIs were measured as −55.3 and −58.2.

Carotid fat density was measured as previously reported by Baradaran et al. [21]. The pericarotid fat density was measured manually. Briefly, the density of the pericarotid fat surrounding the neck internal carotid artery (ICA) was measured in Hounsfield units (HU). Two regions of interest (ROIs) in the pericarotid fat were collected on the same axial slice showing the luminal narrowing (Figure 2) were collected. The ROIs were placed in the fat surrounding the ICA showing maximum luminal narrowing. The ROIs were drawn carefully to include only fat density (fat density was confirmed by negative HUs). To exclude the artery wall and surrounding structures, the ROIs were placed at least 1 mm from the wall of the carotid artery.

Carotid fat density was evaluated by a neurosurgeon blinded to the clinical data. A reader reviewed the radiological findings a second time 4 weeks later, blinded to their first reading, for intra-rater reliability.

### 2.4. MR Plaque Imaging to Evaluate Plaque Composition

Plaque composition was evaluated by MR plaque imaging within 2 days (before or after) CTA. In all patients, the plaque composition was evaluated using a 1.5 T or 3.0 T MR imaging scanner (Magnetom Vision; Siemens, Erlangen, Germany). To characterize the plaque, axial and long-axis images of the carotid artery were obtained from the T1 Sampling Perfection with Application-optimized Contrast using different flip-angle evolutions (SPACE) and time of flight (TOF). The target was decided as the area with the highest degree of stenosis. The 3D T1-weighted SPACE technical parameters were as follows: repetition time/echo time [TR/TE], 500/23 ms; variable flip angle; echo-train length, 32; field of view (FOV), 250 mm × 240 mm; voxel size, 1.3 mm × 1.0 mm × 1.0 mm; GeneRalized Autocalibrating Partial Parallel Acquisition (GRAPPA), 2×; and Spectral Attenuated Inversion Recovery (SPAIR) fat suppression. In addition, 3D TOF MRA was performed in the axial plane using the following imaging sequences: FOV, 220 mm/87.5%; TR, 23 ms; TE, 7.00 ms; slice thickness, 1.2 mm. The T1-weighted imaging parameters were as follows: FOV, 200 mm/100%; TR, 500 ms; TE, 11 ms; and slice thickness, 4 mm. The plaque analysis was measured manually. A signal intensity > 150% of that of the muscle adjacent to the plaque was identified as hyperintense. Hyperintense signals on both T1-weighted and TOF images were judged as IPH, as reported previously [22].

### 2.5. Measurement of Expansive Remodeling Rate

The expansive remodeling ratio was evaluated by the long-axis MRI T1 weighted plaque images as described by Yoshida et al. [11]. The expansive remodeling ratio was defined as the ratio of the distance between the inner border of the carotid arterial lumen and the outer borders of the plaque to the maximum outside diameter of the ICA distal to the plaque. MR plaque images that were insufficient for the measurement of the plaque muscle ratio and expansive remodeling rate, such as those in which it was difficult to identify the plaque border, were excluded from this study.

### 2.6. Immunohistochemistry (IHC)

A total of 125 CEA specimens were obtained and used for IHC analysis. CEA specimens were fixed using formalin. Decalcification was performed using ethylenediaminetetraacetic acid buffer. The specimens were fixed with 4% formaldehyde and then embedded in paraffin. Finally, 4 mm thick axial slices were made. The section with the culprit lesion was selected for IHC. IHC analysis was used to identify macrophages and intraplaque microvessels in carotid plaques. To evaluate the count of macrophage and microvessel markers, we used CD68 and CD31. Briefly, each section was treated with CD68 (clone PG-M1; 1:100; DAKO, Glostrup, Denmark) and CD31 (rabbit monoclonal, 1:100 dilution; Abcam, Cambridge, UK, ab28364) primary antibodies for 40 min at 24 °C. Then, incubation with the EnVision polymer from the DAKO EnVision + Kit (DAKO Cytomation, Glostrup, Denmark) for 60 min was performed. The diaminobenzidine tetrahydrochloride (DAB) chromogen of a DAB substitute kit (DAKO Cytomation) was used. Then, counterstaining with hematoxylin was performed. Carotid plaques were divided by their anatomical location (shoulder, bottom, or core), as previously described [22]. CD68-positive cells and CD31-positive microvessels were counted in the shoulder using ImageJ software Version 1.54j cell counter tool (National Institutes of Health, Bethesda, MD, USA). This process was performed by a certified neurosurgeon.

### 2.7. Statistics

Continuous data are expressed as means ± standard deviation. Welch’s *t*-test, chi-square test, and Fisher’s exact test were applied to compare continuous and dichotomous variables, respectively. Pearson’s product–moment correlation coefficient was used to examine the relationship between pericarotid fat density and the expansive remodeling ratio. Statistical significance was set at *p* < 0.05. Receiver operating characteristic (ROC) analysis was performed on pericardial fat density to determine the optimal thresholds for separating between the symptomatic and asymptomatic subjects by calculating the area under the curve (AUC). The agreement of pericarotid fat density within the observer was measured with the intraclass correlation coefficient (ICC). All statistical analyses were performed using EZR software, version 1.61 (Saitama Medical Center, Jichi Medical University, Saitama, Japan).

## 3. Results

### 3.1. Patient Characteristics

Among the 232 patients with 258 carotid stenoses included in this study, the mean age was 74.4 ± 8.9 years (range: 54–90 years), and 211 (90.9%) and 21 (9.1%) were male and female, respectively. Of the 258 lesions, 130 (50.4%) were symptomatic and 128 (49.6%) were asymptomatic. The comorbidities included hypertension (171 patients, 73.7%), diabetes mellitus (90 patients, 38.8%), dyslipidemia (122 patients, 52.6%), and coronary artery disease (88 patients, 34.1%). The patient characteristics are presented in Table 1.

### 3.2. Radiological Findings

**Figure 3:**
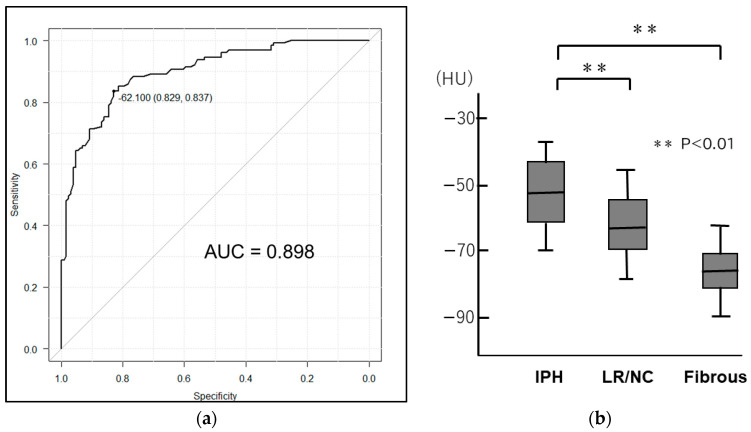
(**a**) ROC analysis identified −62.1 HU as the optimal cutoff for separating symptomatic and asymptomatic lesions, with an AUC of 0.898 (sensitivity: 0.829, specificity: 0.837, 95% confidence interval [CI]: 0.861–0.996). (**b**) The pericarotid fat densities in plaques with IPH, LR/NC, and fibrous plaques were −51.6 ± 10.4 HU, −59.4 ± 12.8 HU, and −74.2 ± 8.4 HU, respectively, and differed significantly among the plaque compositions (one-way analysis of variance [ANOVA], *p* < 0.01).

The mean stenosis degree evaluated by CTA was 70.1 ± 15.2%. Of the 258 carotid stenoses, 163 (63.2%), 63 (24.4%), and 32 (12.4%) were severe, moderate, and mild, respectively. All 32 mild carotid stenoses were symptomatic. Of the 258 carotid stenoses, 94 (36.5%) showed fibrous plaques, while 62 (24.0%) had lipid-rich plaques. The remaining 102 stenoses (39.5%) had IPH plaques. The pericarotid fat density was −63.0 ± 11.1 HU (range −90.5 HU to −41.4 HU). The agreement of pericarotid fat density within the observer was measured with the intraclass correlation coefficient (ICC). The ICC was 0.89. The carotid fat densities were −56.8 ± 10.4 HU and −69.2 ± 11.4 HU in symptomatic and asymptomatic lesions, respectively. ROC analysis identified −62.1 HU as the optimal cutoff for separating symptomatic and asymptomatic lesions, with an AUC of 0.898 (sensitivity: 0.829; specificity: 0.837; 95% confidence interval [CI]: 0.861–0.996) (Figure 3a). However, carotid fat density and age were not correlated (coefficient of correlation: 0.23; 95%CI: 0.19–0.27).

**Figure 4:**
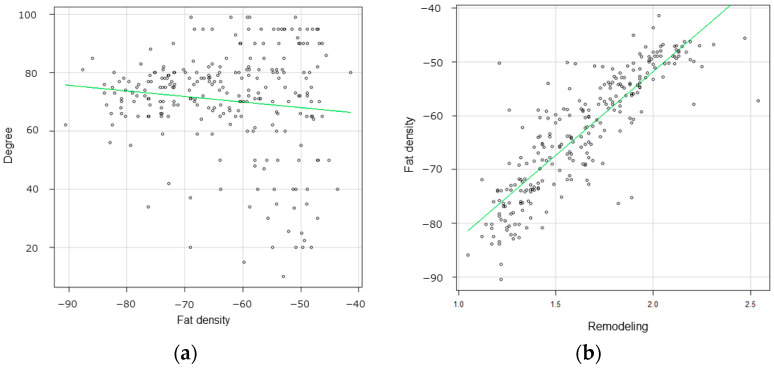
(**a**) Scatterplot shows the relationship between pericarotid fat density and stenosis degree. Pericarotid fat density and the degree of stenosis showed no correlation (coefficient of correlation: −0.12, 95% CI: −0.24 to −0.01). (**b**) The relationship between pericarotid fat density and the expansive remodeling ratio is shown. A positive correlation was observed between carotid fat density and expansive remodeling ratio (co-efficient of correlation: 0.79, 95% CI: 0.72–0.87).

Carotid fat density in symptomatic lesions was significantly higher than that in asymptomatic lesions (*p* < 0.01). Pericarotid fat density varied widely for each plaque composition. The pericarotid fat densities in plaques with IPH, LR/NC, and fibrous plaques were −51.6 ± 10.4 HU, −59.4 ± 12.8 HU, and −74.2 ± 8.4 HU, respectively, and differed significantly among the plaque compositions (one-way analysis of variance [ANOVA], *p* < 0.01) (Figure 3b). Figure 4a shows a scatterplot of the relationship between pericarotid fat density and stenosis degree. Pericarotid fat density and the degree of stenosis showed no correlation (coefficient of correlation: −0.12, 95% CI: −0.24 to −0.01). The expansive remodeling ratio was 1.64 ± 0.41 (range: 1.25–2.54), being 1.41 ± 0.22 and 1.98 ± 0.34 in asymptomatic and symptomatic lesions, respectively. The ratio was significantly higher in symptomatic lesions than in asymptomatic lesions (*p* < 0.01). The relationship between pericarotid fat density and the expansive remodeling ratio is shown in Figure 4b. A positive correlation was observed between carotid fat density and expansive remodeling ratio (coefficient of correlation: 0.79, 95% CI: 0.72–0.87).

### 3.3. Correlation between Pericarotid Fat Density and IHC

**Figure 5:**
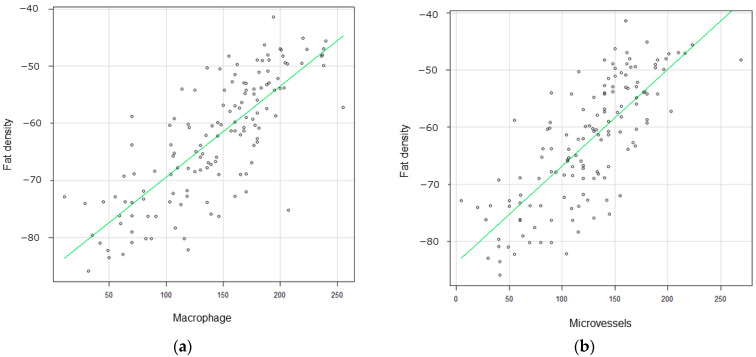
(**a**) Immunohistochemical markers of inflammation in CEA specimens revealed 143.5 ±  61.3/field CD68-positive macrophages in the shoulder area in the carotid plaques. This count correlated well with pericarotid fat density (coefficient of correlation: 0.72, 95% CI: 0.64–0.83). (**b**) The carotid plaques also included 121.2 ± 27.7/field CD31-positive microvessels. This count also correlated well with the pericarotid fat density (coefficient of correlation: 0.70, 95% CI: 0.62–0.80).

**Figure 6:**
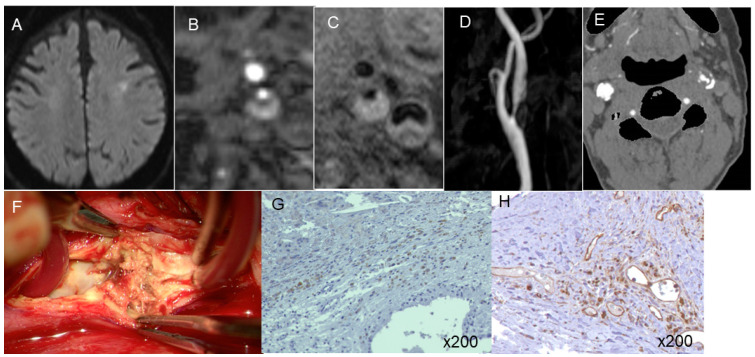
An illustrative case with symptomatic carotid stenosis (**A**). MR plaque imaging showed IPH (**B**,**C**). Expansive remodeling ratio was 1.98 (**D**) and pericarotid fat density was −50.1 HU (**E**). Intraoperative (CEA) photos showed unstable plaque (**F**). Immunohistopathology showed the counts of CD68-positive macrophage (**G**) and CD31-positive microvessels (**H**) were high.

**Figure 7:**
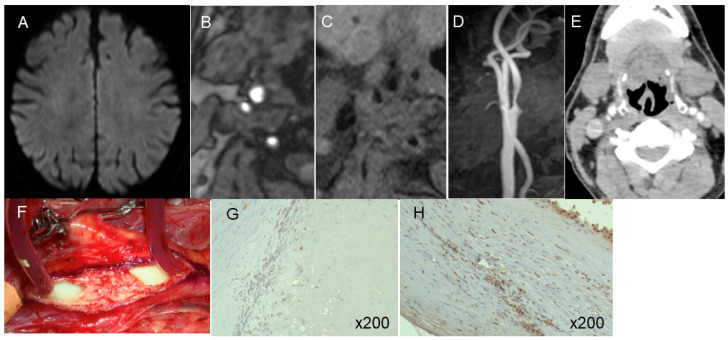
An illustrative case with asymptomatic carotid stenosis (**A**). MR plaque imaging showed fibrous plaque (**B**,**C**). Expansive remodeling ratio was 1.22 (**D**) and pericarotid fat density was −75.1 HU (**E**). Intraoperative (CEA) photos showed stable plaque(**F**). Immunohistopathology showed the counts of CD68 positive macrophage (**G**) and CD31 positive microvessels (**H**) were low.

An evaluation of the relationship between pericarotid fat density and immunohistochemical markers of inflammation in CEA specimens revealed 143.5 ± 61.3/field CD68-positive macrophages in the shoulder area in the carotid plaques. This count correlated well with pericarotid fat density (coefficient of correlation: 0.72, 95% CI: 0.64–0.83) (Figure 5a). The carotid plaques also included 121.2  ±  27.7/field CD31-positive microvessels. This count also correlated well with the pericarotid fat density (coefficient of correlation: 0.70, 95% CI: 0.62–0.80) (Figure 5b). Illustrative cases are presented in Figure 6 and Figure 7.

### 3.4. Radiological Findings in Mild Carotid Stenosis

All 32 patients with mild carotid stenosis had symptomatic carotid stenosis because CEA/CAS was not performed for asymptomatic mild carotid lesions. The stenosis degree was 24.5 ± 15.6%. The plaque composition evaluated by MR included 28 (87.5%) plaques with IPH, 4 (12.5%) LR/NC, and no fibrous plaques. The pericarotid fat density was quite high in mild stenosis (−50.1 ± 8.4 HU). The expansive remodeling ratio was also high (2.08 ± 0.21). The IHC of the CEA specimens showed CD68-positive macrophage and CD31-positive microvessel counts of 170.1 ± 42.3/field and 153.2 ± 42.3/field, respectively. In symptomatic mild carotid stenosis, pericarotid fat density was well correlated with other inflammation factors (CD68-positive macrophages: coefficient of correlation: 0.71, 95% CI: 0.66–0.80; CD31-positive microvessels: coefficient of correlation: 0.74, 95%CI: 0.68–0.83).

## 4. Discussion

This study investigated the association between pericarotid fat density and positive remodeling and inflammatory markers in carotid stenosis. A novel finding of our study was the positive correlation between pericarotid fat density surrounding the carotid artery and a positive remodeling ratio. Furthermore, increased carotid fat density and positive remodeling ratio were associated with plaque vulnerability and symptomatology. However, carotid fat density was not correlated with the degree of stenosis but was correlated with CD68-positive macrophage and CD31-positive microvessel counts. The results of the subgroup analysis suggested that mild carotid stenosis was associated with high pericarotid fat density and an expansive remodeling ratio. IHC revealed high numbers of CD68-positive macrophage and CD31-positive microvessel counts. These data suggest that plaques with mild symptomatic carotid stenosis are accompanied by a marked inflammatory response.

### 4.1. Crosstalk between Pericarotid Fat and the Carotid Artery Wall

Various compartments of fat have local and systemic effects that play crucial roles in health maintenance and disease development. Pericarotid fat releases bioactive molecules, including adipokines, anti- and pro-inflammatory factors, miRNAs, and others. They contribute to the maintenance of vascular homeostasis under physiological conditions [23]. Pericarotid fat tissue attenuation, as measured using CT, is a non-morphological analysis that was initially used for coronary artery plaque [24,25,26]. This is a marker of the mean local vascular inflammation as it reflects histopathological alterations in perivascular adipose tissue in the volume of fat tissue in contact with the vessel.

The role of inflammation in the development and progression of coronary atherosclerosis is firmly established [27]. Crosstalk between the outer vascular layer and the perivascular fat is an important factor in this relationship. Taken together, our results suggest that pericarotid fat density might be a local factor outside the vessel wall that predisposes patients to plaque rupture.

The clinical application of pericarotid fat density in carotid plaques has also been reported. Yu et al. investigated the association between pericarotid fat density and various risk factors of carotid plaques, in which pericarotid fat density was significantly associated with high-risk AHA VI plaque characterization (typical atheroma with a large extracellular lipid core), IPH, and thinning and/or rupture of the fibrous cap [28]. Furthermore, Saba et al. suggested a positive association between pericarotid fat density and contrast plaque enhancement on CTA [29]. This correlation was stronger in symptomatic subjects than in asymptomatic subjects. Our results suggest that pericarotid fat density is correlated with various indirect markers of plaque instability or plaque inflammation. Thus, pericarotid fat density has the potential to be a marker for evaluating carotid plaque vulnerability.

### 4.2. Usefulness of Carotid Fat Density in Clinical Practice

There are several invasive or non-invasive methods used to detect vascular inflammation. As an invasive method, optical coherence tomography has also been used in carotid arteries in order to study atherosclerotic plaque features and to characterize the high-risk plaque in detail [30]. The non-invasive detection of vascular inflammation is a main goal in cardiovascular medicine [31]. To detect residual inflammatory risk is relevant given recent clinical evidence supporting a reduction in risk of unstable coronary plaque with anti-inflammation-based therapy. Various anti-inflammatory interventions have been reported [32,33]. Currently, 18F-FDG PET is one of the most reliable methods for evaluating vascular inflammation and predicting the subsequent risk of ischemic events in comparatively large arteries, such as the carotid artery or aorta. Recently, Zhang et al. suggested the use of integrated 18F-fluorodeoxyglucose positron emission tomography (18F-FDG)/MRI scans to identify distinct risk features in symptomatic and asymptomatic patients [18]. Moreover, Kaczynski et al. suggested that 18F-FDG/MRI characteristics were associated with the culprit carotid vessel in patients with acute neurovascular syndrome [17]. These methods are promising tools for future research. However, controversy remains regarding the application of PET/CT or PET/MRI in clinical practice, mainly because of their high costs. To resolve these issues, the measurement of pericarotid fat density instead of PET may be a useful tool to detect plaque activity in clinical practice. Moreover, while the levels of inflammatory serum markers such as C-reactive protein can help detect inflammatory responses [34,35], they cannot identify specific areas of vascular inflammation. Therefore, a novel, noninvasive method is needed to detect inflammation in carotid plaques.

### 4.3. Pericarotid Fat Density and Positive Remodeling in Mild Carotid Stenosis

The pathogenesis and clinical features of symptomatic mild carotid disease (<50%) have been previously reported. Previously, the management of carotid stenosis relied on the stenosis degree as the primary marker for assessing the high risk of future stroke and the need for intervention [20]. However, the structural factors that define plaque vulnerability may be more useful than the stenosis degree in determining the risk profile and root cause of future ischemic stroke. In the present study, pericarotid fat density was not correlated with the stenosis degree (coefficient of correlation: 0.18). In the clinical setting, Altaf et al. reported that 4 of 22 patients (18%) with symptomatic mild carotid stenosis (30–49% of the patient population) experienced recurrent TIA or stroke despite medical treatment with antiplatelet drugs during a prospective two-year follow-up period. MRI detected the presence of IPH in all four patients, suggesting that plaques with IPH were at high risk for recurrence of cerebrovascular events, regardless of the degree of stenosis and the use of antiplatelet therapy [36]. Nolen et al. suggested that IPH and the total plaque volume are independent risk factors for recurrent ipsilateral ischemic stroke or TIA in patients with mild-to-moderate carotid stenosis [37]. They also concluded that these plaque characteristics could be used to improve current decision-making. While the degree of stenosis is currently the main guiding factor in future stroke risk stratification and long-term therapeutic decision-making, recent evidence suggests that the features of vulnerable plaques offer better prognostic capabilities. This paradigm shift has motivated researchers to seek non-invasive diagnostic tools to image not only the lumen but also the vascular wall and the structural characteristics of plaques [38]. In the present study, pericarotid fat density and the expansive remodeling ratio of symptomatic patients with mild carotid stenosis were consistently high, regardless of the stenosis degree. Therefore, pericarotid fat density may be a novel marker for deciding surgical indications or predicting the risk of ischemic stroke in symptomatic mild carotid stenosis.

This study has several limitations. First, this was a single-center, retrospective study and included a relatively small number of patients. Therefore, a multicenter study with a larger sample size is needed. Second, this was a cross-sectional study; therefore, the immediate application of pericarotid fat density for risk prediction of future stroke in clinical practice is unlikely. Although certain imaging modalities, each with its advantages and disadvantages, can identify mild carotid plaques associated with increased stroke risk, most studies have been cross-sectional, and limited prospective data are available. Therefore, further prospective observational studies are required. The study design might also have resulted in some inherent recall bias, including the lack of a control group of patients who did not undergo CEA/CAS. Therefore, confirmation of our results in larger prospective studies is warranted. Third, this study suffered from a lack of internal control. Finally, this study suffered from the absence of semi-automatic or automatic evaluation and the use of dedicated software for plaque analysis.

## 5. Conclusions

In conclusion, patients with carotid artery stenosis investigated by CTA showed a positive correlation between pericarotid fat density and expansive remodeling ratio. This correlation was stronger in symptomatic patients than in asymptomatic patients. Our results indicate that carotid fat density could be used as an indirect marker of plaque instability in carotid plaques. Pericarotid fat density is a promising diagnostic tool for evaluating plaque inflammation. However, additional multi-center prospective studies are needed to validate these findings and address the limitations of the current study.

## Figures and Tables

**Table 1:** Patient’information, including demographic and radiological data.

	All Lesion	CEA	CAS	*p* Value
	258 lesions	125	133	
Age	74.4 ± 8.9	74.0 ± 8.7	74.8 ± 9.1	0.65
Sex				
Male	237	116	121	0.65
Female	21	9	12	
Symptomatic	130	103	27	<0.01
Asymptomatic	128	30	98	
Comorbidities				
Hypertension	191	89	102	0.32
Diabetes mellitus	90	40	50	0.36
Dyslipidemia	122	60	62	0.9
Coronary artery disease	88	40	48	0.51
Laboratory				
LDL cholesterol (mg/dL)	109.8 ± 20.1	112.3 ± 22.2	106.1 ± 18.6	0.55
HbA1c	6.1 ± 1.1	6.0 ± 1.0	6.2 ± 1.2	0.74
Drug administration				
Hypertension	220	105	115	0.6
Diabetes mellitus	85	39	46	0.6
Dyslipidemia (statin)	128	60	68	0.62
Antiplatelet	165	82	83	0.61
Anticoagulant	22	10	12	0.71
Radiological findings				
% Stenosis	70.1 ± 15.2%	64.7 ± 25.2%	75.8 ± 9.2%	<0.01
Severe (≥70%)	163	55	108	
Moderate (50–69%)	63	40	23	
Mild (<50%)	32	30	2	
Plaque composition				
Fibrous plaques	94	19	75	<0.01
LR/NC	62	34	28	
IPH	102	72	30	

CEA: carotid endarterectomy, CAS: carotid artery stenting, LR/NC: lipid-rich–necrotic core, IPH: intraplaque hemorrhage.

## Data Availability

The data analyzed in the current study are available from the corresponding author upon reasonable request.
